# Cortical Responsiveness to Nociceptive Stimuli in Patients with Chronic Disorders of Consciousness: Do C-Fiber Laser Evoked Potentials Have a Role?

**DOI:** 10.1371/journal.pone.0144713

**Published:** 2015-12-16

**Authors:** Antonino Naro, Margherita Russo, Antonino Leo, Carmela Rifici, Patrizia Pollicino, Placido Bramanti, Rocco Salvatore Calabrò

**Affiliations:** IRCCS Centro Neurolesi Bonino-Pulejo, Messina, Italy; University of British Columbia, CANADA

## Abstract

It has been shown that the presence of Aδ-fiber laser evoked potentials (Aδ-LEP) in patients suffering from chronic disorders of consciousness (DOC), such as vegetative state (VS) and minimally conscious state (MCS), may be the expression of a residual cortical pain arousal. Interestingly, the study of C-fiber LEP (C-LEP) could be useful in the assessment of cortical pain arousal in the DOC individuals who lack of Aδ-LEP. To this end, we enrolled 38 DOC patients following post-anoxic or post-traumatic brain injury, who met the international criteria for VS and MCS diagnosis. Each subject was clinically evaluated, through the coma recovery scale-revised (CRS-R) and the nociceptive coma scale-revised (NCS-R), and electrophysiologically tested by means of a solid-state laser for Aδ-LEP and C-LEP. VS individuals showed increased latencies and reduced amplitudes of both the Aδ-LEP and C-LEP components in comparison to MCS patients. Although nearly all of the patients had both the LEP components, some VS individuals showed only the C-LEP ones. Notably, such patients had a similar NCS-R score to those having both the LEP components. Hence, we could hypothesize that C-LEP generators may be rearranged or partially spared in order to still guarantee cortical pain arousal when Aδ-LEP generators are damaged. Therefore, the residual presence of C-LEP should be assessed when Aδ-LEP are missing, since a potential pain experience should be still present in some patients, so to properly initiate, or adapt, the most appropriate pain treatment.

## Introduction

In contrast to comatose patients, who lack of both wakefulness and awareness, individuals suffering from Vegetative State (VS) are awake but unaware of the environment and cannot purposefully respond to stimuli, whilst patients affected by Minimally Conscious State (MCS) are awake but limitedly aware and may show some purposeful behaviors [1–2].

Pain perception in chronic disorder of consciousness (DOC) represents a controversial issue, since there is growing evidence concerning the presence of residual pain experience even in some VS individuals [3–6]. Taking into account that such patients have a strongly limited repertoire of communication, and that the inability to communicate could not exclude a possible pain experience, the issues of nociception and pain in such individuals are of ethic and clinical importance, especially concerning a proper diagnosis and an adequate pain treatment.

Laser evoked potentials (LEP) are extensively used in pain study, since the laser stimulation can selectively activate the nociceptive pathways [7]. Laser stimulation typically evokes several LEP components, reflecting the activity of multiple cortical assemblies within different cortical areas (including primary and secondary somatosensory cortices, insula, and anterior cingulate cortex) that process either nociceptive or non-nociceptive inputs [8–9]. The N1P1 complex reflects an early stage of sensory processing at unaware level, whereas the N2P2 wave is related to the stimulus saliency, independently from the nociceptive nature of the incoming stimulus [8,10–12].

Although the Aδ-fiber LEP (Aδ-LEP) amplitudes and latencies have been put in relation with the intensity of nociceptive pain [12], it has been shown that LEP depend on non-specific neural pathways within the so-called pain-matrix, so that LEP characteristics cannot be considered as a marker of pain-related cognitive processes [8,13–14], but only a sign of a relevant-stimulus dependent arousal [8–15].

Interestingly, recent LEP studies have shown reliable cortical responses following nociceptive stimuli in VS and MCS individuals [10–11], regardless of the preservation of non-nociceptive somatosensory evoked potentials. Such issues may suggest that “the cortical awareness toward pain stimulus may be a basal function for survival in state of vegetative autonomy, despite the absence of evident motor reaction to nociceptive inputs”. Therefore, nociceptive stimuli may be processed even in severe DOC patients [10–11].

Nevertheless, whereas Aδ-LEP have been studied in DOC patients [10–11], the assessment of C-fiber LEP (C-LEP) has not been yet performed in DOC individuals. C-LEP have been shown to be generated by C-fiber activation through laser stimuli with specific characteristics, but they probably share common cortical generators with Aδ-LEP [16–18].

We hypothesized that the VS patients who lack of Aδ-LEP could show a cortical pain arousal, as demonstrated by C-LEP preservation, and they therefore might experience pain. Therefore, we tested this hypothesis by assessing the presence and the characteristics of C-LEP elicited by means of a solid-state laser device in a sample of DOC patients lacking of Aδ-LEP.

## Materials and Methods

The study was conducted in accordance with the Declaration of Helsinki and was approved by Ethic Committee of IRCCS Centro Neurolesi “Bonino-Pulejo” all the HC subjects and the legal guardian of the DOC patients gave written informed consent before any study-related procedures were performed. Moreover, the families of the patients gave written informed consent to publish the potentially identifying case details.

### Subjects

We enrolled 38 DOC subjects affected by anoxic or traumatic brain injury, who met the international criteria for VS and MCS diagnosis [1–2]. The exclusion criteria were: cutaneous or systemic disease contraindicating LEP execution; critical conditions (e.g. mechanical ventilation, hemodynamic instability); peripheral nervous system damage; brainstem lesions. As control group, we enrolled 15 healthy age-matched individuals (HC). We summarized the detailed clinic and demographic characteristics of DOC patients in Table 1. All of the patients and the HC had previously performed an Aδ-LEP study, according to the standard procedures of Cruccu and coworkers [19–20].

**Table 1:** Shows the clinic-demographic characteristics and the Aδ-LEP and C- LEP amplitude and latency values (individual mean of each laser stimulation run ±SD, SD of the two laser stimulation runs, and *group averages±SD*). Patients showing only the N2P2 CLEP are marked in bold.

et	age	gen	dd	CRS	NCS	Aδ-LEP												C-LEP											
						N1(ms)		SD	N1(μV)		SD	N2(ms)		SD	N2(μV)		SD	N1(ms)		SD	N1(μV)		SD	N2(ms)		SD	N2(μV)		SD
**MCS (n = 15)**																													
A	64	F	14	17	8	127±16	130±12	2	1±0.5	1±0.5	0.005	172±22	170±12	2	11±1	12±1	0.7	213±27	222±16	7	3.3±0.5	3.4±0.5	0.03	306±40	314±49	5	6.6±1	6±1	0.4
A	58	F	13	17	7	138±18	137±11	1	3.2±0.5	3±0.5	0.1	188±24	185±16	3	6.1±1	6.4±1	0.3	213±27	210±21	2	5.4±0.5	5.7±0.5	0.2	365±47	361±28	3	4.3±0.5	4.1±0.5	0.1
A	60	M	13	17	6	168±22	165±11	3	6.4±0.5	6.6±0.5	0.1	185±24	192±12	5	5.4±1	5.2±1	0.1	181±23	177±28	3	4.5±0.5	4.7±0.5	0.2	275±36	264±48	8	3±0.5	3.1±0.5	0.1
T	48	M	33	17	5	141±18	145±18	3	1.1±0.5	1±0.5	0.1	231±30	237±13	4	5.4±1	5.4±1	0.1	216±28	207±36	6	3.2±0.5	3.4±0.5	0.1	347±44	343±60	2	3.2±0.5	3.3±0.5	0.1
T	37	M	26	17	5	152±20	148±18	3	2.2±0.5	2±0.5	0.1	190±25	194±16	3	1±0.5	1.1±1	0.1	226±29	234±15	6	5.5±0.5	5.4±0.5	0.1	275±36	279±28	3	3.3±0.5	3.1±0.5	0.2
T	66	F	23	17	9	178±23	171±14	3	2.1±0.5	2.2±0.5	0.01	183±24	182±14	1	2.1±0.5	2.1±1	0.02	247±32	247±14	0.5	6.7±0.5	6.1±0.5	0.4	308±39	301±22	5	3.3±0.5	3.2±0.5	0.1
A	62	M	30	16	9	127±16	125±10	2	5.2±0.5	5±0.5	0.02	164±21	167±19	2	7.3±1	7.6±1	0.2	181±23	179±14	1	3.6±0.5	3±0.5	0.4	291±38	296±53	4	3.3±0.5	3.1±0.5	0.1
A	61	M	18	16	5	131±17	135±13	3	1±0.5	1.1±0.5	0.003	200±26	201±15	1	17±1	14±1	2	209±27	220±17	8	4.4±0.5	4.4±0.5	0.04	241±31	244±19	1	1.4±0.5	3.2±0.5	1.3
A	75	M	24	16	5	114±15	118±14	3	1±0.5	1.2±0.5	0.05	159±21	152±15	5	6.4±1	6.4±1	0.001	276±36	275±17	0.5	3.2±0.5	3.2±0.5	0.02	312±40	302±15	8	4±0.5	4±1	0.03
T	60	M	32	16	6	122±16	124±18	2	2.2±0.5	2±0.5	0.01	131±17	134±19	2	7.6±1	7.7±1	0.1	315±41	309±25	4	3.4±0.5	3.4±0.5	0.01	275±36	225±15	0.5	5.3±1	5.5±1	0.1
T	57	F	33	16	5	133±17	132±18	1	4.4±0.5	4.1±0.5	0.1	179±23	180±13	1	14±2	13±1	0.7	218±28	207±17	7	5.8±0.5	5.7±0.5	0.1	347±45	346±20	2	2.2±0.5	2±0.5	0.1
T	68	F	15	16	6	116±15	119±14	3	2.1±0.5	2.2±0.5	0.04	185±24	185±14	0.5	10±2	10±1	0.2	227±28	237±12	7	3.2±0.5	3.0±0.5	0.1	278±36	286±49	5	5.3±0.5	5.1±0.5	0.1
T	59	M	17	16	9	113±15	115±13	2	5.4±0.5	5±0.5	0.1	149±19	145±12	3	4.1±1	4.1±1	0.02	218±28	208±12	7	6±0.5	5.4±0.5	0.5	237±31	227±21	7	3±0.5	3.1±0.5	0.1
A	66	F	29	15	9	109±14	106±19	2	3.3±0.5	3.1±0.5	0.1	185±24	191±16	4	10±1	10±1	0.4	253±33	253±14	0.5	5.9±0.5	5.8±0.5	0.02	255±33	262±42	4	2.1±0.5	2.1±0.5	0.1
T	57	F	32	15	8	128±17	131±11	2	2.2±0.5	2±0.5	0.1	188±24	188±14	0.5	8.2±1	7.5±1	0.5	237±31	224±19	9	4.3±0.5	4.3±0.5	0.04	324±42	328±60	4	4.4±1	4.4±0.5	0.02
	*60±9*		*23±8*	*16±1*	*7±2*	*130±19*	*131±17*	*2±1*	*2*.*9±1*.*8*	*2*.*8±1*.*8*	*0*.*03±0*.*05*	*178±25*	*179±27*	*2±2*	*7*.*6±4*.*1*	*7*.*4±3*.*7*	*0*.*1*	*235±34*	*233±35*	*4±3*	*4*.*6±1*.*2*	*4*.*5±1*.*2*	*0*.*1*	*290±36*	*291±40*	*4±2*	*3±1*	*4±1*	*0*.*2±0*.*3*
**VS (n = 23)**																													
A	62	F	15	7	2	167±30	166±12	1	4.4±0.5	4.1±0.6	0.1	231±42	230±25	1	13±2	11±0.6	1	344±63	348±44	3	2.1±0.5	2.1±0.5	0.05	448±82	446±54	1	3±1	2±0.5	1
A	64	F	25	7	2	170±31	165±11	3	4.1±1	4.1±1	0.02	215±39	216±10	1	9±2	13±0.5	3	314±57	316±41	2	1±0.5	2.1±0.5	0.8	410±75	405±44	4	3±1	3.5±0.5	0.4
T	62	M	19	7	2	129±24	125±19	3	2±0.5	4±0.5	1	179±33	189±11	7	6±1	8±0.5	1	442±81	344±35	2	1±0.5	1.1±0.5	0.1	*596±109*	592±56	3	7±1	3±0.5	3
**T**	**50**	**M**	**11**	**7**	**2**	**157±29**	**159±17**	**2**	**5±1**	**2±0.5**	**2**	**NA**	**NA**	**NA**	**NA**	**NA**	**NA**	**423±77**	**326±32**	**2**	**1.1±0.5**	**1±0.5**	0.04	**531±97**	**532±73**	**1**	**6±1**	**6.6±0.5**	**0.4**
T	67	M	16	7	2	147±27	143±11	2	4±1	5±0.5	1	217±40	213±19	3	6.3±1	6.5±0.5	0.1	461±66	463±35	2	3.1±0.5	1.1±0.5	1.4	382±70	405±18	16	7±1	6±0.5	1
**A**	**59**	**M**	**32**	**6**	**2**	**133±24**	**136±13**	**3**	**5±1**	**4±0.5**	**1**	**NA**	**NA**	**NA**	**NA**	**NA**	**NA**	**314±57**	**319±29**	**3**	**1±0.5**	**3.2±0.5**	1.5	**398±73**	**408±32**	**7**	**2±0.5**	**8±0.5**	**4**
A	58	M	17	6	2	158±29	155±11	3	4±1	5±0.5	1	266±59	267±35	1	7±1	9±0.5	1	397±72	393±34	4	2.1±0.5	1.1±0.5	0.7	446±81	442±16	3	3±1	2±0.5	1
T	80	F	18	6	2	155±28	153±18	3	1±0.5	4±0.5	2	249±38	247±13	1	10±2	6±0.5	3	485±59	492±56	5	2.1±0.5	2±0.5	0.03	382±80	387±55	4	6±1	3±0.5	2
A	70	F	29	5	2	140±26	143±11	2	4±1	1±0.5	2	NA	NA	NA	NA	NA	NA	345±63	343±20	2	2±0.5	2.1±0.5	0.1	NA	NA	NA	NA	NA	NA
**T**	**59**	**F**	**27**	**5**	**1**	**158±29**	**154±13**	**3**	**5±1**	**4±0.5**	**1**	**NA**	**NA**	**NA**	**NA**	**NA**	**NA**	**323±55**	**338±49**	**4**	**2.2±0.5**	**2.1±0.5**	0.1	**361±66**	**363±66**	**2**	**5±1**	**6±1**	**0.1**
A	64	F	16	4	1	116±21	119±18	2	1±0.5	5±0.5	3	NA	NA	NA	NA	NA	NA	472±59	464±27	8	2.2±0.5	2.2±0.5	0.01	NA	NA	NA	NA	NA	NA
T	63	F	24	4	1	179±33	180±14	1	5±1	1±0.5	3	NA	NA	NA	NA	NA	NA	400±73	404±19	3	2.1±0.5	2.1±0.5	0.01	NA	NA	NA	NA	NA	NA
A	28	M	16	3	1	145±26	145±10	0.5	6±1	5±0.5	1	NA	NA	NA	NA	NA	NA	466±81	470±59	3	3.3±0.5	2.2±0.5	0.8	NA	NA	NA	NA	NA	NA
T	48	F	32	3	1	169±31	169±11	0.5	4±1	7±0.5	2	177±32	182±14	4	7±1	6±0.5	1	443±81	434±55	7	3.1±0.5	3.3±0.5	0.2	555±61	557±63	1.4	8±1	8.2±1	0.1
A	45	M	17	2	0	148±27	149±17	1	1±0.5	4±0.5	2	NA	NA	NA	NA	NA	NA	456±83	467±53	8	3.2±0.5	3±0.5	0.1	NA	NA	NA	NA	NA	NA
A	43	F	20	2	0	161±29	162±13	1	5±1	1±0.5	3	NA	NA	NA	NA	NA	NA	358±65	371±10	9	3.1±0.5	3.1±0.5	0.01	NA	NA	NA	NA	NA	NA
T	58	F	33	2	1	159±29	160±15	1	4±1	5±0.5	1	NA	NA	NA	NA	NA	NA	406±74	398±28	6	2.1±0.5	3.2±0.5	0.8	NA	NA	NA	NA	NA	NA
A	48	F	26	2	0	155±28	154±18	1	4.1±1	4.1±1	0.02	NA	NA	NA	NA	NA	NA	409±78	405±21	3	3.1±1	2.1±0.5	0.7	NA	NA	NA	NA	NA	NA
A	47	F	26	6	0	155±28	158±19	2	4.1±1	4.1±1	0.02	130±24	135±13	4	2.2±0.5	2.1±0.5	0.1	453±79	457±19	3	3±1	3±0.5	0.01	382±70	408±10	18	5.3±1	5.1±0.5	0.13
**T**	**46**	**M**	**15**	**5**	**2**	**155±30**	**158±12**	**2**	**4±1**	**4±0.5**	**0**	**NA**	**NA**	**NA**	**NA**	**NA**	**NA**	**406±80**	**408±17**	**2**	**3.3±1**	**3.3±0.5**	**0.01**	**370±68**	**371±43**	**1**	**5.4±1**	**5±0.5**	**0.3**
**T**	**66**	**M**	**7**	**5**	**0**	**155±27**	**161±14**	**4**	**4.4±0.5**	**4.1±0.5**	**0.1**	**NA**	**NA**	**NA**	**NA**	**NA**	**NA**	**440±80**	**449±66**	**6**	**3±1**	**3.3±0.5**	**0.2**	**358±65**	**362±56**	**3**	**5.5±1**	**5.5±0.5**	**0.03**
T	44	M	11	7	0	156±21	154±11	1	4.3±0.5	4.3±0.5	0.04	227±40	131±17	3	6±1	NA	NA	444±81	444±72	0.5	3.3±1	3±0.5	0.2	345±63	343±11	2	5.4±1	5.4±0.5	0.01
**A**	**48**	**M**	**13**	**6**	**2**	**156±26**	**160±10**	**3**	**4.3±0.5**	**4.3±0.5**	**0.04**	**NA**	**NA**	**NA**	**NA**	**NA**	**NA**	**448±82**	**447±70**	**1**	**3±1**	**3.2±0.5**	**0.1**	**333±61**	**348±21**	**10**	**5±1**	**5.4±0.5**	**0.3**
	*55±11*		*20±7*	*5±2*	*1±1*	*153±13*	*152±13*	*2±1*	*4±1*	*4±1*	*0*.*1±0*.*8*	*207±41*	*199±45*	*3±2*	*7±3*	*8±3*	*1±1*	*415±51*	*406±54*	*4±2*	*2±1*	*2*.*2±1*	*0*.*1*	*418±80*	*422±77*	*5±5*	*5±2*	*5±2*	*1*.*1±1*.*3*
**HC (n = 15)**																													
	65	F				118±11	119±11	0.5	13±1	9±1	3	171±16	166±15	4	11±1	9±0.5	1	232±21	242±29	7	5±0.5	26±0.5	15	255±23	248±18	4	10±1	13±2	2
	60	M				152±17	153±17	0.5	10±1	11±1	1	223±22	129±14	4	11±1	12±05	0.1	187±17	187±17	0.5	8±1	9±1	0.1	312±28	313±28	0.5	16±1	17±1	1
	61	M				136±13	138±10	1	8±1	12±1	3	165±15	166±13	1	9±1	11±0.5	1	234±21	238±11	5	6±1	5±0.5	1	266±21	270±11	3	19±2	10±0.5	6
	55	M				158±24	159±15	1	12±1	10±1	1	187±17	288±13	1	12±1	11±0.5	1	182±16	191±10	7	6±1	8±0.5	1	291±27	293±14	2	18±2	16±0.5	1
	61	M				122±11	135±17	10	9±1	8±1	1	126±12	128±14	1	14±1	9±0.5	4	251±21	254±15	4	4±0.5	6±0.5	1	304±28	309±12	3	12±1	19±0.5	5
	65	M				145±13	150±15	4	8±1	12±1	3	248±23	241±12	5	12±1	14±0.5	1	224±20	226±23	3	5±0.5	6±0.5	1	298±18	300±63	2	17±2	19±0.5	1
	61	F				119±11	126±12	5	10±1	9±1	1	212±19	215±17	2	9±1	13±0.5	3	139±13	135±10	2	9±1	4±0.5	4	229±21	222±12	5	11±5	11±0.5	1
	63	F				150±12	152±15	1	10±1	8±1	1	164±15	168±17	3	30±3	12±0.5	1	201±18	208±10	5	7±1	5±0.5	1	350±32	352±28	1	8±1	16±0.5	6
	55	F				130±15	126±15	3	8±1	10±1	1	176±16	177±13	1	12±1	9±0.5	2	162±15	159±17	1	8±1	9±0.5	0.1	354±32	355±28	1	6±1	11±0.5	4
	56	F				110±10	119±17	7	9±1	10±1	1	168±15	163±17	4	18±2	28±0.5	7	171±15	168±15	2	6±1	7±0.5	1	245±22	250±14	4	16±1	8±0.5	6
	65	F				131±12	132±17	0.5	9±1	8±1	1	202±18	205±16	2	29±3	13±0.5	4	147±13	150±17	2	3±0.5	9±0.5	4	355±32	351±16	3	11±1	6±0.5	4
	56	M				119±10	119±16	0.5	6±1	9±1	2	152±14	159±17	4	23±2	19±0.5	4	158±14	167±11	6	4±0.5	6±0.5	1	253±20	252±49	1	11±1	15±0.5	3
	59	F				162±15	160±17	1	10±1	9±1	1	206±19	210±15	3	33±3	28±0.5	11	183±17	181±17	1	6±1	3±0.5	2	217±29	218±12	1	12±1	10±0.5	1
	55	M				143±13	143±12	0.5	10±1	6±1	3	187±17	181±17	4	11±1	21±0.5	7	227±21	229±17	4	6±1	4±0.5	1	317±23	313±17	5	8±1	11±0.5	2
	65	F				134±12	133±17	1	9±1	10±1	1	167±15	167±17	0.5	21±2	33±0.5	15	169±15	178±13	6	4±0.6	6±0.5	1	253±23	252±10	1	13±1	11±0.5	1
	*60±4*					*132±16*	*137±14*	*5±5*	*9±2*	*9±2*	*2±1*	*178±30*	*176±35*	*3±2*	*17±8*	*16±8*	*4±4*	*182±30*	*177±34*	*6±5*	*6±2*	*7±5*	*2±4*	*276±65*	*258±85*	*2±1*	*13±4*	*13±4*	*3±2*

*Legend*: A: post-anoxic brain-injury; age in years; CRS-R: coma recovery scale-revised; dd: disease duration in months; et: etiology; gen: gender; MCS: minimally conscious state; NCS-R: nociception coma scale-revised; sd: standard deviation; T: post-traumatic brain-injury; VS: vegetative state.

### Clinical assessment

DOC individuals (23 VS and 15 MCS) were clinically evaluated by two neurologists, skilled in DOC diagnosis, through the JFK Coma Recovery Scale-Revised (CRS-R). This scale is a reliable and standardized tool, which integrates neuropsychological and clinical assessment, and includes the current diagnostic criteria for coma, VS and MCS, allowing the clinician to assign the patient to the most appropriate diagnostic category. Thus, the CRS-R is considered as an appropriate measure for characterizing level of consciousness and for monitoring neurobehavioral function recovery [21].

Pain perception was specifically evaluated by means of the Nociception Coma Scale-Revised (NCS-R) [22] that has been developed for assessing pain in severely brain-injured patients, and allows a better specification of the conscious behavioral patterns linked to pain experience in MCS and VS. This tool consists in the observation of motor, verbal, and facial responses to painful stimulation. The total score ranges from zero to 9. A cut-off of 4 has been proposed to suggest an aware pain perception.

### Laser stimulation set-up

LEP were recorded at bed and over a reclining armchair in a quiet and mild-lighted room in DOC patients and HC individuals, respectively. All measures concerning laser-safety were observed (protective goggles, earplugs). Aδ-LEP were previously recorded in either HC or DOC patients. We used a Neodymium:Yttrium-Aluminium-Perovskite laser (Nd:YAP) (wavelength 1.34μm, pulse duration 2-20ms, maximum energy 7J, 4mm beam diameter) with fiber-optic guidance (Electronic Engineering, Florence, Italy). A red helium–neon (He–Ne) laser, confocal with the infrared beam, was used to visually indicate the irradiated skin area of the right trigeminal maxillary branch region, close to the nasus-genius sulcus. In each individual, we employed a laser stimulation intensity of 60-80mJ/mm^2^ that induced a Visual Analogic Score of at least 4/10 in the HC. Such intensity produced clear and stable evoked components in all of the HC individuals, and was therefore used throughout the entire experimental procedure. The mean stimulation intensity employed in HC individuals was used to evoke LEP in the DOC sample. We delivered two trains of 30 laser pulses, with a 5-minute inter-train interval. In order to avoid habituation phenomena and skin overheating and damage, the delivery frequency was 0.1±0.025Hz and the stimulator, whose handle was held perpendicularly to skin surface, was slightly shifted over the skin. During the stimulation, HC did not perform any mental task (neutral condition) [16], whereas DOC patients practiced the CRS-R arousal protocol [23] before the LEP session.

In order to record C-LEP, the laser stimulation was carried out according to Bragard and co-workers protocol [24] that obtained the direct isolation of C-LEP from tiny cutaneous surfaces by means of a CO_2_ laser. The laser stimuli (wavelength 1.34μm, pulse duration 10ms, 4mm beam diameter) were directed to the skin area of the right trigeminal maxillary branch region, close to the nasus-genius sulcus, but a thin aluminium disk, drilled with calibrated holes (~0.15mm^2^) was interposed just above the skin surface [24]. Stimulus intensity was individually adapted in the HC individuals, so that sensations reported ranged from “barely detectable” to “slight pain”. Such intensity produced clear and stable evoked components in all of the HC individuals, and was, therefore, used throughout the entire experimental procedure. The mean stimulation intensity applied in HC individuals was employed to evoke C-LEP in the DOC sample.

### LEP recording

LEP were recorded from three Ag-AgCl scalp-surface electrodes (put on Fp2, Cz, and T3). The reference electrode was put on the nose and the ground on Fpz. Eye movements and blinks were monitored by an electrode above the right eyebrow. Electrode impedance was kept ≤5kΩ. The time-analysis was set at 2s, with a pre-analysis period of 100ms. Signals were filtered at 0.3-70Hz, sampled at 250Hz through a Synergy-Medelec System (Tecnomed, Pescara, Italy), and stored on a PC for off-line analysis. Trials contaminated by ocular or muscle artifacts were automatically excluded from the analysis through an artifact-rejection system that excluded all runs containing transient signals exceeding ±65μV in any recording channel (including the electro-oculogram) from the average. In the HC group, we identified three LEP components, defined in terms of estimated topography and latency values, i.e the N2P2 (Cz-nose), the N1 (T3-nose), and the P1 (Fp2-nose). The time-windows were determined after having analyzed the grand-average of all the LEP, and then applied in each individual analysis. Thus, we measured the baseline-peak amplitude (μV) of N1 and N2 waves, and the latency (ms) of N2 and N1 (in addition, we calculated the N1P1 by computing an offline bipolar montage T3-Fp2, where phase reversal is evident) [17,25], in either the single runs or the average of the two runs. Latencies were determined by using a modified box-plot method known as the median rule [26].

### Statistical analysis

Latencies and amplitudes of the LEP components were analyzed at their scalp sites through a one-way analysis of variance (ANOVA), with *group* (three levels: HC, VS, and MCS) as between-subject factor. Cases without LEP were labeled as missing data. The Greenhouse-Geisser method was used if necessary to correct the degrees of freedom [27]. A p-value <0.05 after correction was accepted as statistically significant. Conditional on a significant F-value, *post-hoc* analysis (Tukey's honestly significant difference -THSD) was performed to explore the strength of the main effects and the patterns of interaction between experimental factors. All data are given as means± standard deviation (SD). We also calculated a two-tailed bivariate Pearson’s correlation coefficient between the clinical (CRS-R and NCS-R) and electrophysiological parameters (latencies and amplitudes of Aδ-LEP and C-LEP components).

## Results

**Fig 1:**
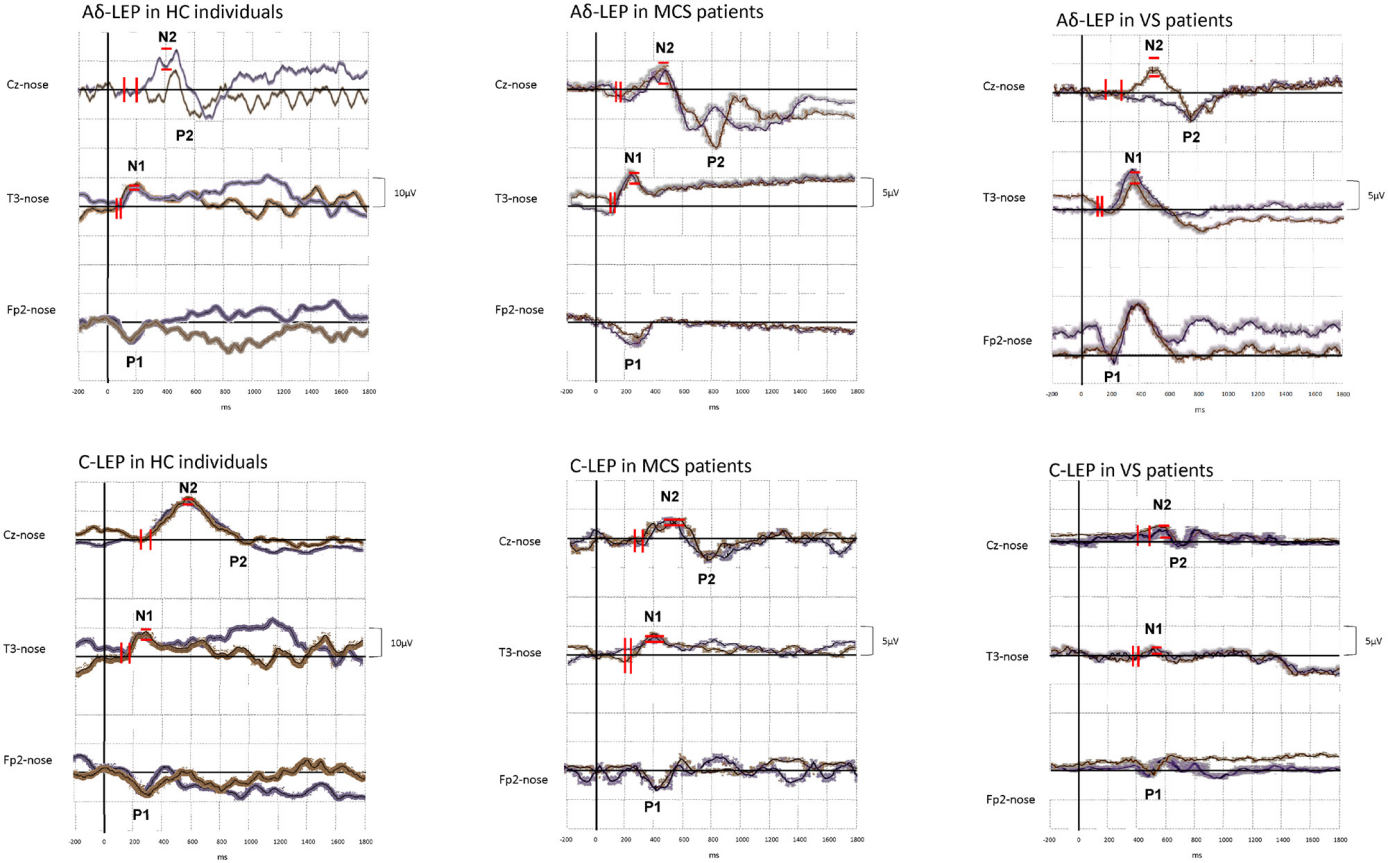
Shows the grand averages in the time-domain (the two thick waveforms represent the two pulse-train–runs, the shady areas express the inter-subject variability) of Aδ-LEP and C-LEP at electrode Cz (N2P2), T3 (N1) and Fp2 (P1). Amplitude negative values are plotted up. The vertical and horizontal black lines indicate the time point of the laser stimulation and the zero line, respectively. The vertical and horizontal red bars refer to the standard deviation concerning LEP latency and amplitude, respectively.

We did not report any side effects during and after the stimulation protocol. In Table 1, we summarize the single-subject values of LEP latencies and amplitudes in the two runs of laser stimulation, the standard deviation of LEP amplitude and latencies between the two laser stimulation runs for each subject, the group values of LEP latencies and amplitudes, and the clinical and demographic characteristics of HC and DOC patients. In Fig 1, we reported the two averaged waveforms at each electrode in the two groups of DOC patients, concerning either Aδ-LEP or C-LEP.

The Aδ-LEP stimulation set up induced both the N1P1 and N2P2 components, as well as the C-LEP paradigm produced the ultra-late LEP waves. Either the HC or the DOC patients showed reliable evoked responses, since the LEP waveforms were track-by-track stable within each laser stimulation run, and the two consecutive averaged waveforms were almost superimposable at either individual or group level (Fig 1). Similarly, the individual LEP values showed a run-by-run low variability (i.e. no more than 3 times the mean run-by-run SD) (Table 1).

All of the participants showed the N1P1 component of both Aδ-LEP and C-LEP. All of the HC and the MCS patients showed the Aδ-LEP N2P2 component, whereas this was missing in 13 VS individuals (Table 1). Interestingly, six out of these patients showed the C-LEP N2P2 component. Hence, the VS individuals showed three distinct N2P2 LEP patterns: i) both the Aδ-LEP N2P2 and C-LEP (10 individuals); ii) only the C-LEP N2P2 (6 individuals); and iii) neither the Aδ-LEP N2P2 nor the C-LEP N2P2 (7 individuals). Notably, none of the patients showed the Aδ-LEP N2P2 without the C-LEP N2P2.

We found higher LEP amplitudes and less delayed LEP latencies in HC than DOC patients. On the other hand, LEP latencies were significantly higher in VS than MCS individuals, whereas LEP amplitudes were almost superimposable. The statistically significant inter-group differences are summarized in Table 2. The other parameters (age, disease duration, etiology, and gender) were not significantly different among the three groups, and the LEP parameters did not correlate with the clinical scale scores.

**Table 2:** Resumes the one-way ANOVA findings (*group* effect) and the *post-hoc* THSD data concerning LEP parameters. NS stands for non-significant.

	one-way ANOVA		*post-hoc* THSD		
	F_(2,100)_	p	HC/MCS	HC/VS	MCS/VS
**Aδ-LEP**					
N1 latency	4.9	0.01	NS	0.02	0.01
N1 amplitude	75	<0.001	<0.001	<0.001	NS
N2 latency	3.9	0.03	NS	0.02	0.01
N2 amplitude	11	<0.001	<0.001	0.003	NS
**C-LEP**					
N1 latency	67	<0.001	0.01	<0.001	<0.001
N1 amplitude	19	<0.001	0.001	<0.001	0.002
N2 latency	29	<0.001	NS	<0.001	<0.001
N2 amplitude	42	<0.001	<0.001	<0.001	NS

## Discussion

To the best of our knowledge, this is the first study investigating C-LEP in DOC patients. Our findings agree with previous reports showing an increased latency of Aδ-LEP in DOC patients (more in VS than MCS individuals) without any significant amplitude inter-group difference [10–11]. The preservation of N2P2 Aδ-LEP may suggest a residual nociceptive cortical processing in severe brain-injured patients, even when other sensory evoked potentials are missing [10–11]. Therefore, a potential pain experience should be taken into account in such patients.

It is worthy to note that it is not so easy to record C-LEP, as reported by the international guidelines [28–29]. Therefore, a first critical question is whether the evoked responses we obtained may or not reliably represent C-LEP. Indeed, there are two main problems in C-LEP recording: i) the preceding Aδ-LEP may hinder the following C-LEP; and ii) the extremely low range of conduction speed of the unmyelinated fibers limits the necessary synchronization of the input to produce a clear signal from the scalp (in fact, C-LEP have been mainly investigated in facial territories). There are two main methods to record C-LEP: i) Bragard et al. [24] and Opsommer et al. [30] employed a laser beam passing through a grid with micro-spots, without any substantial Aδ-LEP interference; and ii) Iannetti et al. [13] and Cruccu et al. [31] used very large and low-energy laser beams, directly focused on skin, thus stimulating warmth receptors of the skin overlying the spine.

We used the Bragard’s approach (i.e. the micro-spot technique) [24], since mechano/heat-sensitive A-fiber nociceptors (AMHs) are less numerous than mechano/heat-sensitive C-fiber nociceptors (CMHs), and therefore the micro-spot technique reduces the probability of activating AMHs. Instead, the large-beam and low-intensity method [13,31] allows a selective activation of the warmth C-fiber receptors, which have a lower threshold than AMHs and CMHs. Nevertheless, AMHs are more sparse on face but have larger receptive fields than CMHs and warmth C-fiber receptors. Hence, the probability of activating AMHs could be the same with or without the spatial restriction provided by the grid [32]. However, in the present study we used a solid-state laser, which has well-known different properties in comparison to the gas one, with the former showing a shorter wavelength and a deeper penetration power [14,33]. Instead, gas laser has a greater energy dissipation [34–35] when has to activate the deep nociceptors (i.e. at the dermo-epidermal junction, approximately 100–500μm). Moreover, the short pulse duration of solid-state laser reduces the time for CMHs activation and generates a more synchronous afferent volley [36], leading to more synchronized brain evoked potentials [37]. In addition, AMHs in glabrous skin respond with a long latency of several seconds to sustained heat stimulus and can be sensitized dramatically, whereas CMHs innervating glabrous skin do not share these properties [38–39]. Hence, there is a bulk of data attesting to the suitability of solid-state lasers in activating CMHs [31,40].

In our study, we at first registered C-LEP in HC individuals, identifying clear and stable LEP components, with a significantly low intra-subject and inter-subject variability. In addition, the two averaged trains of stimuli were overimposable. In DOC patients, the evoked responses were more delayed in latency and smaller in amplitude in comparison to HC, but they still showed a low intra-subject variability, and a similar inter-train superimposability. Therefore, taking into account the aforementioned methodological discussion, we believe that the responses we obtained reliably expressed the C-LEP. Moreover, although several hundred stimuli were necessary in order to record C-LEP in a previous study [24], we could use a smaller number of laser stimuli because of differences in our methodology, including the laser type employed (solid-state vs. gas) and the site of stimulation (face vs. hand).

Notably, 13 VS patients did not show any Aδ-LEP N2P2 response, whereas previous studies reported a high-level of Aδ-LEP preservation [10–11]. Such discrepancy between our findings and de Tommaso’s data [10–11] may depend on either the larger sample we studied, or the aforementioned methodological issues.

By a quantitative point of view, C-LEP latencies were significantly higher in the VS than the MCS individuals, whereas there were no significant inter-group differences concerning C-LEP N2P2 amplitude. Nonetheless, our LEP patterns did not significantly correlate with the clinical assessment, without any relation between pain arousal and the awareness level, according to previous works [10–11]. Moreover, some VS patients showed very small LEP amplitude, even for the C-LEP amplitude range. This may depend on the variability of P2 waveforms we observed, which could have somehow influenced the baseline-peak amplitude values of N2 waves.

Interestingly, six out of the 13 VS patients lacking of Aδ-LEP N2P2 component showed clear C-LEP N2P2 waves. Moreover, such patients had a clinical picture, NCS-R and CRS-R score, similar to the VS patients showing both the Aδ-LEP and C-LEP N2P2 components. Hence, we may argue that the cortical arousal towards nociceptive stimuli could be guaranteed in those patients lacking of Aδ-LEP by a reshuffle or a residual preservation of cortical C-LEP generators. In our opinion, such findings raise an important question concerning the functional role of the C-LEP generators, since we did not observe any patients showing only Aδ-LEP N2P2 without C-LEP N2P2 waves. Although previous source analysis studies have shown that Aδ-LEP and C-LEP cortical generators may share similar circuits (even if related to different Aδ/C-fiber activation) [9], they independently process (either in series or in parallel) [31] some features of the afferent inputs (e.g. abrupt vs. slow-changing stimuli) [41]. Taking into account the functional differentiation of LEP generators, a substitute role of C-LEP N2P2 generators could be due to an over-activation of the slow-conducting medial pain-system (that oversees the nociceptive-related reflexive and affective responses) [41–42], which in turn depends on a brain injury-induced limbic and subcortical hyperconnectivity [43]. In addition, a selective modulation of the regional cortical excitability, an enlargement of the receptive fields, and the effects of cortical deafferentation could be taken into account in an attempt to explain a C-LEP generator preservation or reorganization [44–47]. Moreover, we could speculate about a strong C-LEP network stability in reason of an older phylogenetic origin [48], and a functional switch to a fixed “in-parallel” or “in-series” processing of nociceptive inputs [49].

The main limitations in our study are to be considered the small number of EEG channels we employed (since we were not able to use a full-EEG cap), and the non-homogeneous gender and age matching of our sample.

In conclusion, our work suggests that DOC patients may somehow show a residual cortical responsiveness to nociceptive stimuli. Indeed, C-LEP preservation could indicate the presence of a residual pain arousal even in the VS patients who do not show Aδ-LEP. Therefore, a possible pain experience should be carefully assessed in each VS patient so to properly initiate, or adapt, the most appropriate treatment.

## References

[1] The Multi-Society Task Force on PVS. Medical aspects of the persistent vegetative state. New Eng J Med 1994, 330:1499–1508. 7818633 10.1056/NEJM199405263302107

[2] Giacino J, Ashwal S, Childs N, Cranford R, Jennett B, Katz DI, et al The minimally conscious state: Definition and diagnostic criteria. Neurology 2002, 58:349–353. 11839831 10.1212/wnl.58.3.349

[3] Kassubek J, Juengling FD, Els T, Spreer J, Herpers M, Krause T, et al Activation of a residual cortical network during painful stimulation in long-term post-anoxic vegetative state: a ^15^O-H_2_O PET study. J Neurol Sci 2003, 212: 85–91. 12810004 10.1016/s0022-510x(03)00106-0

[4] Zanatta P, Messerotti Benvenuti S, Baldanzi F, Bosco E. Pain-related middle-latency somatosensory evoked potentials in the prognosis of post anoxic coma: a preliminary report. Min Anestesiol 2012, 78:749–756. 22337155

[5] Boly M, Faymonville ME, Peigneux P, Lambermont B, Damas F, Luxen A, et al Cerebral processing of auditory and noxious stimuli in severely brain injured patients: differences between VS and MCS. Neuropsychol Rehab 2005, 15:283–289. 10.1080/09602010443000371 16350972

[6] Laureys S, Faymonville ME, Peigneux P, Damas P, Lambermont B, Del Fiore G, et al Cortical processing of noxious somatosensory stimuli in the persistent vegetative state. Neuroimage 2002, 17:732–741. 12377148

[7] Treede RD, Magerl W, Baumgärtner U. Laser-evoked potentials for assessment of nociceptive pathways in humans. Toward a rational experimental and clinical use. Pain Forum 1998, 7:191–195.

[8] Mouraux A, Iannetti GD. Nociceptive laser-evoked brain potentials do not reflect nociceptive-specific neural activity. J Neurophysiol 2009, 101:3258–3269. 10.1152/jn.91181.2008 19339457

[9] Garcia-Larrea L, Frot M, Valeriani M. Brain generators of laser-evoked potentials: from dipoles to functional significance. Neurophysiol Clin 2003, 33:279–292. 14678842 10.1016/j.neucli.2003.10.008

[10] de Tommaso M, Navarro J, Ricci K, Lorenzo M, Lanzillotti C, Colonna F, et al Pain in prolonged disorders of consciousness: laser evoked potentials findings in patients with vegetative and minimally conscious states. Brain Inj 2013, 27:962–972. 10.3109/02699052.2013.775507 23789870

[11] de Tommaso M, Navarro J, Lanzillotti C, Ricci K, Buonocunto F, Livrea P, Lancioni GE. Cortical responses to salient nociceptive and not nociceptive stimuli in vegetative and minimal conscious state. Front Hum Neurosci 2015, 9:17 10.3389/fnhum.2015.00017 25688200 PMC4310288

[12] Iannetti GD, Hughes NP, Lee MC, Mouraux A. Determinants of laser-evoked EEG responses: pain perception or stimulus saliency? J Neurophysiol 2008, 100:815–828. 10.1152/jn.00097.2008 18525021 PMC2525705

[13] Iannetti GD, Truini A, Romaniello A, Galeotti F, Rizzo C, Manfredi M, et al Evidence for a specific spinal pathway for the sense of warmth in humans. J Neurophysiol 2003, 89:562–570. 12522202 10.1152/jn.00393.2002

[14] Mouraux A, Plaghki L. Are the processes reflected by late and ultra-late laser evoked potentials specific of nociception? Suppl Clin Neurophysiol 2006, 59:197–204. 16893112 10.1016/s1567-424x(09)70031-5

[15] Ronga I, Valentini E, Mouraux A, Iannetti GD. Novelty is not enough: laser-evoked potentials are determined by stimulus saliency, not absolute novelty. J Neurophysiol 2013, 109:692–701. 10.1152/jn.00464.2012 23136349 PMC3567386

[16] Opsommer E, Weiss T, Miltner WHR, Plaghki L. Scalp topography of ultralate (C-fiber) evoked potentials following thulium YAG laser stimuli to tiny skin surface areas in humans. Clin Neurophysiol 2001, 112:1868–1874. 11595145 10.1016/s1388-2457(01)00622-8

[17] Valeriani M, Restuccia D, Le Pera D, De Armas L, Maiese T, Tonali P. Attention-related modifications of ultra-late CO_2_ laser evoked potentials to human trigeminal nerve stimulation. Neurosci Lett Supp 2002, 329:329–333. 10.1016/s0304-3940(02)00671-7 12183042

[18] Wang AL, Mouraux A, Liang M, Iannetti GD. Stimulus Novelty, and Not Neural Refractoriness, Explains the Repetition Suppression of Laser-Evoked Potentials. J Neurophysiol 2010, 104:2116–2124. 10.1152/jn.01088.2009 20592123

[19] Cruccu G, Aminoff MJ, Curio G, Guerit JM, Kakigi R, Mauguiere F, et al Recommendations for the clinical use of somatosensory-evoked potentials. Clin Neurophysiol 2008, 119:1705–1019. 10.1016/j.clinph.2008.03.016 18486546

[20] Cruccu G, Sommer C, Anand P, Attal N, Baron R, Garcia-Larrea L, et al EFNS guidelines on neuropathic pain assessment: revised 2009. Eur J Neurol 2010, 17:1010–1018. 10.1111/j.1468-1331.2010.02969.x 20298428

[21] Gerrard P, Zafonte R, Giacino JT. Coma Recovery Scale-Revised: evidentiary support for hierarchical grading of level of consciousness. Arch Phys Med Rehab 2014, 95:2335–2341. 10.1016/j.apmr.2014.06.018 25010536

[22] Chatelle C, Majerus S, Whyte J, Laureys S, Schnakers C. A sensitive scale to assess nociceptive pain in patients with disorders of consciousness. JNNP 2012, 83:1233–1123. 10.1136/jnnp-2012-302987 22906615

[23] Kalmar K, Giacino JT. The JFK Coma Recovery Scale-Revised. Neuropsychol Rehabil 2005, 15:454–460. 16350986 10.1080/09602010443000425

[24] Bragard D, Chen CAN, Plaghki L. Direct isolation of ultra-late (C-fiber) evoked brain potentials by CO2 laser stimulation of tiny cutaneous surface areas in man. Neurosci Lett 1996, 209:81–84. 8761987 10.1016/0304-3940(96)12604-5

[25] Treede RD, Lorenz J, Baumgärtner U. Clinical usefulness of laser-evoked potentials. Neurophysiol Clin 2003, 33:303–314. 14678844 10.1016/j.neucli.2003.10.009

[26] Letham B, Raij T. Statistically robust measurement of evoked response onset latencies. Neurosci Methods 2011, 194:374–379. 10.1016/j.jneumeth.2010.10.016 PMC3010493 20974175

[27] Geisser S, Greenhouse SW. An Extension of Box's Results on the Use of the F Distribution in Multivariate Analysis. Ann Math Statist 1958, 29:885–891.

[28] Cruccu G, Sommer C, Anand P, Attal N, Baron R, Garcia-Larrea L, et al EFNS guidelines on neuropathic pain assessment: revised 2009. Eur J Neurol 2010, 17:1010–1018. 10.1111/j.1468-1331.2010.02969.x 20298428

[29] Haanpää M, Attal N, Backonja M, Baron R, Bennett M, Bouhassira D, et al NeuPSIG guidelines on neuropathic pain assessment. Pain. 2011, 152:14–27. 10.1016/j.pain.2010.07.031 20851519

[30] Opsommer E, Masquelier E, Plaghki L. Determination of nerve conduction velocity of C-fibers in humans from thermal thresholds to contact heat (thermode) and from evoked brain potentials to radiant heat (CO2 laser). Neurophysiol Clin 1999, 29:411–422. 10587951 10.1016/S0987-7053(00)87265-2

[31] Cruccu G, Pennisi E, Truini A, Iannetti GD, Romaniello A, Le Pera D, et al Unmyelinated trigeminal pathways as assessed by laser stimuli in humans. Brain 2003, 126:2246–2256. 12847077 10.1093/brain/awg227

[32] Truini A, Galeotti F, Haanpaa M, Zucchi R, Albanesi A, Biasiotta A, et al Pathophysiology of pain in post-herpetic neuralgia: a clinical and neurophysiological study. Pain 2008, 140:405–410. 10.1016/j.pain.2008.08.018 18954941

[33] Bromm B, Treede R. CO2 laser radiant heat pulses activate C nociceptors in man. Pflug Arch 1983, 399:155–156. 10.1007/BF00663913 6417623

[34] Spiegel J, Hansen C, Treede RD. Clinical evaluation criteria for the assessment of impaired pain sensitivity by thulium-laser evoked potentials. Clin Neurophysiol 2000, 111:725–735. 10727924 10.1016/s1388-2457(99)00297-7

[35] Plaghki L, Mouraux A. How do we selectively activate skin nociceptors with a high power infrared laser? Physiology and biophysics of laser stimulation. Neurophysiol Clin 2003, 33:269–277. 14678841 10.1016/j.neucli.2003.10.003

[36] Perchet C, Godinho F, Mazza S, Frot M, Legrain V, Magnin M, et al Evoked potentials to nociceptive stimuli delivered by CO2 or Nd:YAP lasers. Clin Neurophysiol 2008, 119:2615–2622. 10.1016/j.clinph.2008.06.021 18848804

[37] Iannetti GD, Leandri M, Truini A, Zambreanu L, Cruccu G, Tracey I. A nociceptor response to laser stimuli: selective effect of stimulus duration on skin temperature, brain potentials and pain perception. Clin Neurophysiol 2004, 115:2629–2637. 15465452 10.1016/j.clinph.2004.05.023

[38] Campbell JN, Meyer RA. Sensitization of unmyelinated nociceptive afferents in the monkey varies with skin type. J Neurophysiol 1983, 49:98–110. 6298377 10.1152/jn.1983.49.1.98

[39] Lamotte RH, Thalhammer JG, Robinson CJ. Peripheral neural correlates of magnitude of cutaneous pain and hyperalgesia: a comparison of neural events in monkey with sensory judgement in human. J Neurophysiol 1983, 50:1–26. 6875640 10.1152/jn.1983.50.1.1

[40] Hu L, Cai MM, Xiao P, Luo F, Iannetti GD. Human Brain Responses to Concomitant Stimulation of A and C Nociceptors. J Neurosci 2014, 34:11439–11451. 10.1523/JNEUROSCI.1355-14.2014 25143623 PMC6615513

[41] Garcia Larrea L, Convers P, Magnin M, André Obadia N, Peyron R, Laurent B, et al Laser evoked potential abnormalities in central pain patients: the influence of spontaneous and provoked pain. Brain 2002, 125:2766–2781. 12429603 10.1093/brain/awf275

[42] Vogt BA, Sikes RW. The medial pain system, cingulate cortex, and parallel processing of nociceptive information. Progr Brain Res 2000, 122:223–235. 10.1016/s0079-6123(08)62141-x 10737061

[43] Di Perri C, Bastianello S, Bartsch AJ, Pistarini C, Maggioni G, Magrassi L, et al Limbic hyperconnectivity in the vegetative state. Neurology 2013, 8:1417–1424. 10.1212/WNL.0b013e3182a43b78 24049132

[44] Lenz FA. The thalamus and central pain syndromes: human and animal studies In: Case KL, ed. Pain and Central Nervous System Disease. New York: Raven Press, 1991.

[45] Bennett GJ. Neuropathic pain In: Wall PD, Melzack R, ed. Textbook of pain. Edinburgh: Churchill Livingstone, 1994.

[46] Weng HR, Lee JI, Lenz FA, Schwartz A, Vierck C, Rowland L, Dougherty PM. Functional plasticity in primate somatosensory thalamus following chronic lesion of the ventral lateral spinal cord. Neurosci 2000, 101:393–401. 10.1016/s0306-4522(00)00368-7 11074162

[47] Wu Q, Garcia-Larrea L, Mertens P, Beschet A, Sindou M, Mauguiere F. Hyperalgesia with reduced laser evoked potentials in neuropathic pain. Pain 1999, 80:209–214. 10204733 10.1016/s0304-3959(98)00206-1

[48] Montagna W. Cutaneous Innervation In: Montagna W, ed. Proceedings of the Brown University Symposium on the Biology of Skin. New York: Pergamon Press, 1959.

[49] Magerl W, Ali Z, Ellrich J, Meyer RA, Treede RD. C- and A-delta fiber components of heat-evoked cerebral potentials in healthy human subjects. Pain 1999, 82:127–137. 10467918 10.1016/S0304-3959(99)00061-5

